# Senescence gives insights into the morphogenetic evolution of anamniotes

**DOI:** 10.1242/bio.025809

**Published:** 2017-05-12

**Authors:** Éric Villiard, Jean-François Denis, Faranak Sadat Hashemi, Sebastian Igelmann, Gerardo Ferbeyre, Stéphane Roy

**Affiliations:** 1 Department of Stomatology, Faculty of Dentistry, Université de Montréal, Montréal, Québec H3T 1J4, Canada; 2 Department of Biochemistry and Molecular Medicine, Faculty of Medicine, Université de Montréal, Montréal, Québec H3T 1J4, Canada

**Keywords:** Axolotl, Evolution, Morphogenesis, Pronephros, Senescence, Zebrafish

## Abstract

Senescence represents a mechanism to avoid undesired cell proliferation that plays a role in tumor suppression, wound healing and embryonic development. In order to gain insight on the evolution of senescence, we looked at its presence in developing axolotls (urodele amphibians) and in zebrafish (teleost fish), which are both anamniotes. Our data indicate that cellular senescence is present in various developing structures in axolotls (pronephros, olfactory epithelium of nerve fascicles, lateral organs, gums) and in zebrafish (epithelium of the yolk sac and in the lower part of the gut). Senescence was particularly associated with transient structures (pronephros in axolotls and yolk sac in zebrafish) suggesting that it may play a role in the elimination of these tissues. Our data supports the notion that cellular senescence evolved early in vertebrate evolution to influence embryonic development.

## Abstract (precis)

**Summary:** We report the presence of senescent cells in several transient structures in developing amphibian and teleost fish, suggesting novel mechanisms of morphogenesis that appeared early in vertebrate evolution.

## INTRODUCTION

Cellular senescence has been, until recently, linked to aging and as a means to prevent aberrant cellular proliferation. Cells that become senescent stop proliferating and secrete multiple cytokines, growth factors and extracellular matrix remodeling enzymes. This senescence-associated secretory phenotype (SASP) mediates multiple physiological effects of senescent cells (Tchkonia et al., 2013). Although senescence per se is not cell death, senescent cells *in vivo* are cleared by phagocytosis (Xue et al., 2007). The initial view of senescence as a means to eliminate premalignant cells has been radically changed by the groups of Serrano (Munoz-Espin et al., 2013) and Keyes (Storer et al., 2013) who independently published that cellular senescence is also important for embryonic development in mice. More recently, Yun et al. found that senescent cells were transiently present in regenerating limbs of axolotls where they are eliminated by macrophages (Yun et al., 2015). The same group also published a paper looking at senescence during development in axolotls and *Xenopus* and they showed that TGF-β is implicated in maintaining senescence in these organisms (Davaapil et al., 2017). To determine how widespread developmental senescence is, we examined senescence in multiple sections of axolotl and in zebrafish embryonic development.

## RESULTS AND DISCUSSION

**Fig. 1.**
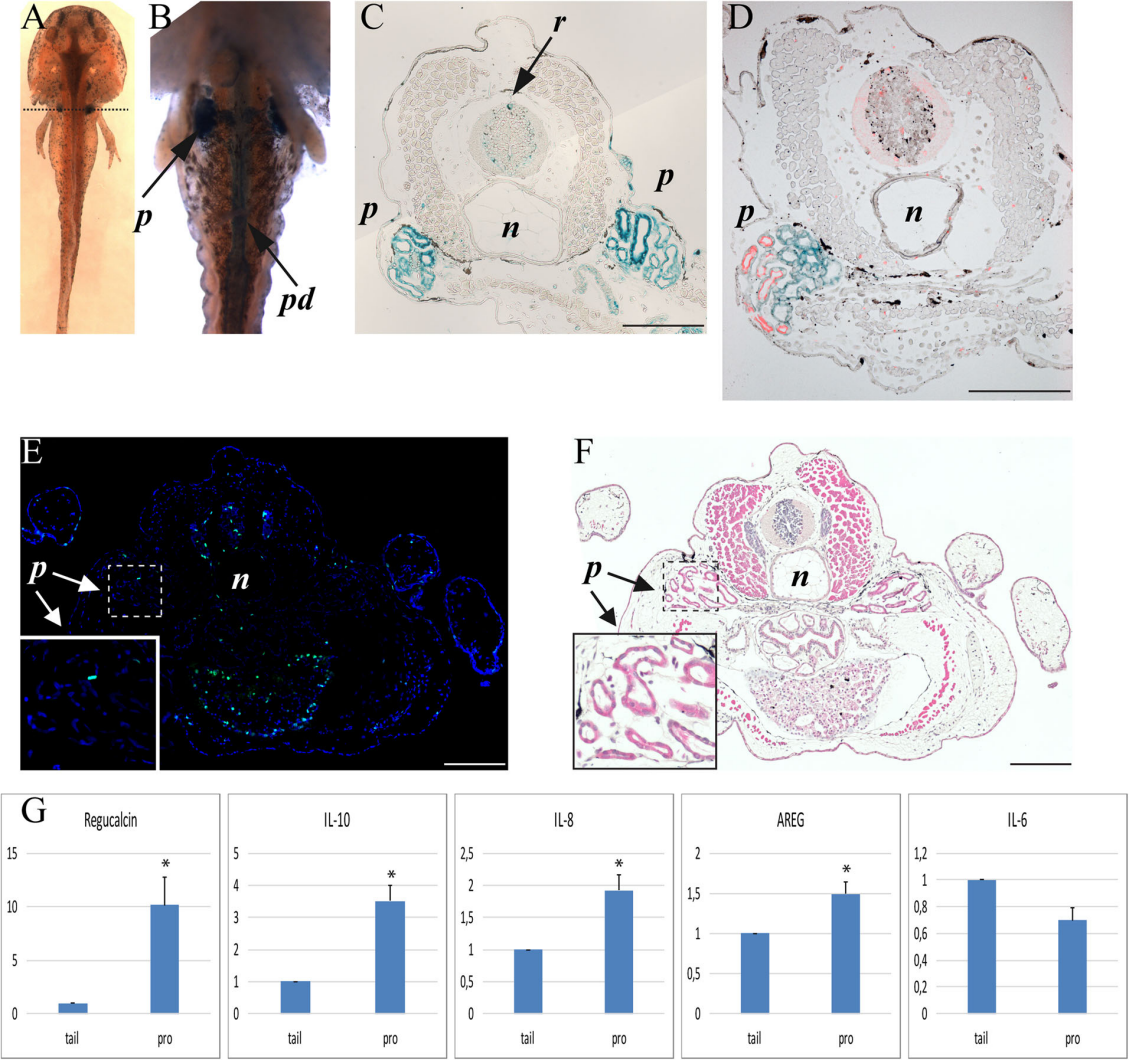
**Axolotl stage 50, pronephric area.** (A) Dorsal view of wholemount SaβG staining (dotted line showing transversal section level). (B) Ventral view of wholemount SaβG staining. (C-F) Transversal section of pronephric area. (C) SaβG staining. (D) SaβG staining (blue) and phospho-*Erk1/2* (red). (E) BrdU staining (green) and DAPI staining (blue). (F) H&E staining of panel E. (G) qRT-PCR measuring pronephros-specific gene regucalcin and SASP genes IL-10, IL-8, AREG and IL-6 in pronephric area versus tail tissue caudal to the pronephros. *P≤0.05 (paired *t*-test), mean±s.e.m. (normalized using GAPDH, *n*=4). *p*, pronephros; *pd*, pronephric duct; *n*, notochord; *r*, roof plate. Composite images are shown. Scale bars: 200 µm.

Developing axolotl and zebrafish embryos were fixed in glutaraldehyde and stained for the senescence-associated β-galactosidase (SaβG) activity. We detected a strong SaβG activity in the pronephros of axolotls (Fig. 1A-D). SaβG activity was also detected along the pronephric ducts from the pronephros all along until the bladder (Fig. 1B). Some primitive fishes retain pronephros all their life but in salamanders it is present transiently and is replaced by the mesonephros as they reach maturity (Hildebrand, 1988). Senescence in mammals is often associated with the presence of phospho-Erk (Deschenes-Simard et al., 2013), a characteristic also present in the axolotl pronephros (Fig. 1D). In addition, the SaβG-positive pronephros is negative for BrdU incorporation which indicates that these cells are not proliferating (Fig. 1E,F). We also isolated total RNA from sections of axolotl embryos containing the pronephros and sections from the tail, which did not stain significantly for the presence of senescent cells and compared the expression of various markers associated with senescence. First we tested the enrichment of pronephric RNA by assessing the levels of the pronephric-specific gene regucalcin (Sato et al., 2000). We can see in Fig. 1G that regucalcin is significantly higher in the RNA preps containing the pronephros. Other genes associated with SASP were assessed as interleukin (IL)-10, -8, -6 and Amphiregulin (AREG) (Coppé et al., 2008). We observe that IL-10, IL-8 and AREG are significantly higher in the RNA prep enriched for pronephros transcripts and IL-6 is not significantly different (Fig. 1G). This is interesting as IL-6 is strongly linked to inflammation while IL-10 is an anti-inflammatory cytokine. This implies that developmental senescence is not associated with inflammation.

**Fig. 2.**
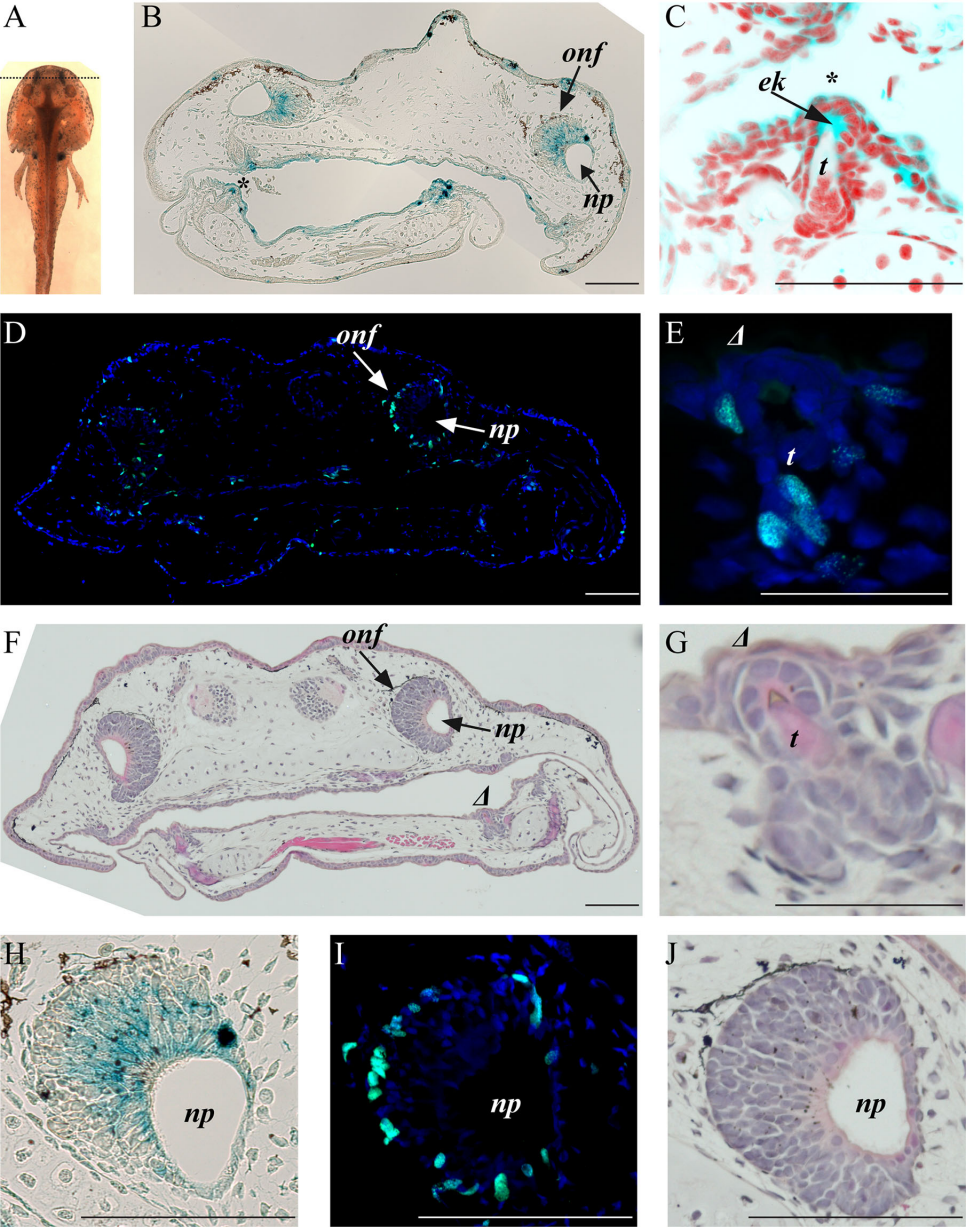
**Axolotl stage 50, rostral area (mouth/nasal pit).** (A) Dorsal view showing wholemount SaβG staining (dotted line represent transversal section level). (B) Rostral transverse section (SaβG staining). (C) Tooth, magnified from panel B, marked with * [SaβG staining in blue and nuclei in red (derived from DAPI stain which is blue that was converted to red in Photoshop to be able to overlay with the blue from SaβG)]. (D) Rostral transverse section, BrdU staining (green) and DAPI staining (blue). (E) Tooth magnified from panel D, marked with *Δ*, BrdU staining (green) and DAPI staining (blue). (F) Rostral transverse section, H&E staining of panel D. (G) Tooth, magnified from panel F, marked with *Δ*, H&E staining of panel E. (H) Olfactory nerve fascicule from panel B, SaβG staining. (I) Olfactory nerve fascicule from panel D, BrdU staining. (J) Olfactory nerve fascicule from panel F, H&E staining. *onf*, olfactory nerve fascicle; *np*, nasal pit; *ek*, enamel knot; *t*, tooth. Composite images are shown. Scale bars: 200 µm.

**Fig. 3.**
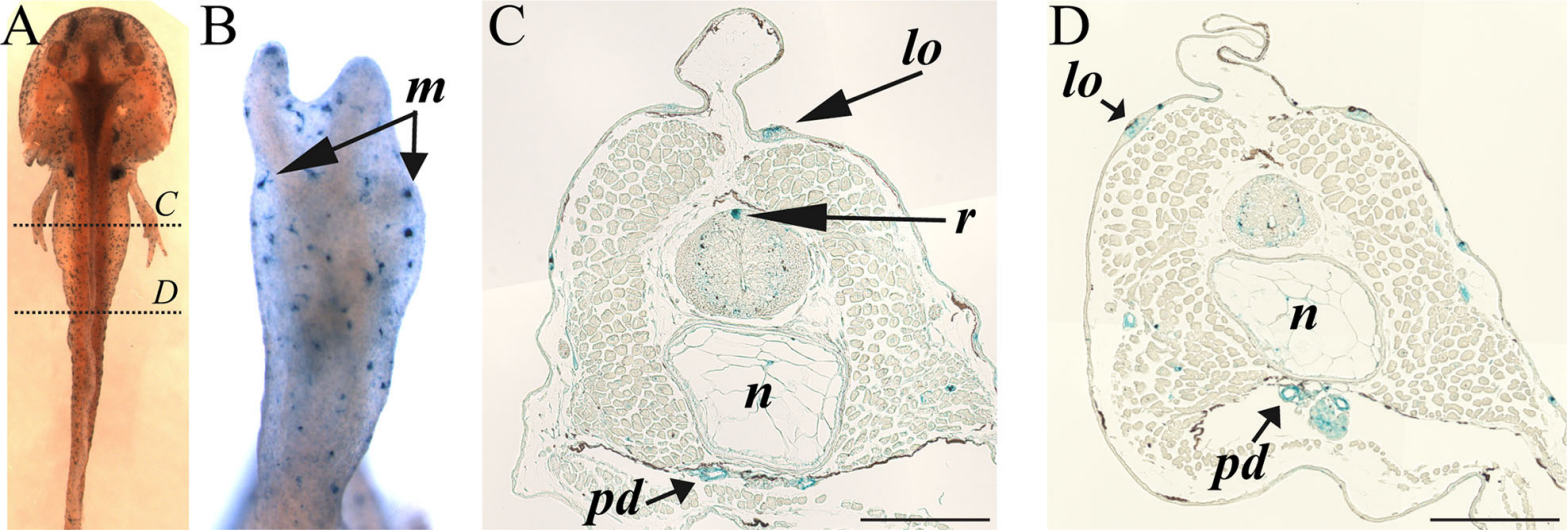
**Axolotl stage 50, mid-body area, SaβG staining.** (A) Dorsal view of wholemount SaβG staining with dotted lines showing subsequent transversal section levels. (B) Forelimb SaβG staining. (C) Mid-body transverse section. (D) Caudal transverse section. *lo*, lateral organ; *r*, roof plate; *n*, notochord; *pd*, pronephric duct. Composite images are shown. Scale bars: 200 µm.

A second strong staining area for senescent cells was detected in the olfactory nerve fascicles in the nasal pit area corresponding to the olfactory epithelium (Fig. 2A,B,H). Again, SaβG-positive cells in the olfactory epithelium do not incorporate BrdU (Fig. 2I). The BrdU-positive cells in the olfactory nerve fascicles are observed in the outside edge where no SaβG can be detected (Fig. 2H,I). If the SaβG assay was allowed to proceed for an extra 2-4 h at 37°C, other structures appeared as possibly containing senescent cells. Interestingly, SaβG-positive and BrdU-negative cells at the point where teeth are erupting through the gums (Fig. 2C,E,G) are reminiscent of the mammalian enamel knot. Cells comprising the enamel knot do not proliferate and end up being eliminated by apoptosis to allow the teeth to grow through the gum epithelium (Matalova et al., 2004). The enamel knot is a signaling center, similar to the apical ectodermal ridge (aer) of the amniote limb bud. Storer et al. demonstrated that aer cells are positive for senescence even though they also display apoptosis (Francis et al., 2005; Storer et al., 2013). Finally, we observed SaβG activity associated with some skin cells (Fig. 3A,B), in the roof plate of the developing spinal cord (Fig. 3C) and in the lateral organs (Fig. 3C,D).

**Fig. 4.**
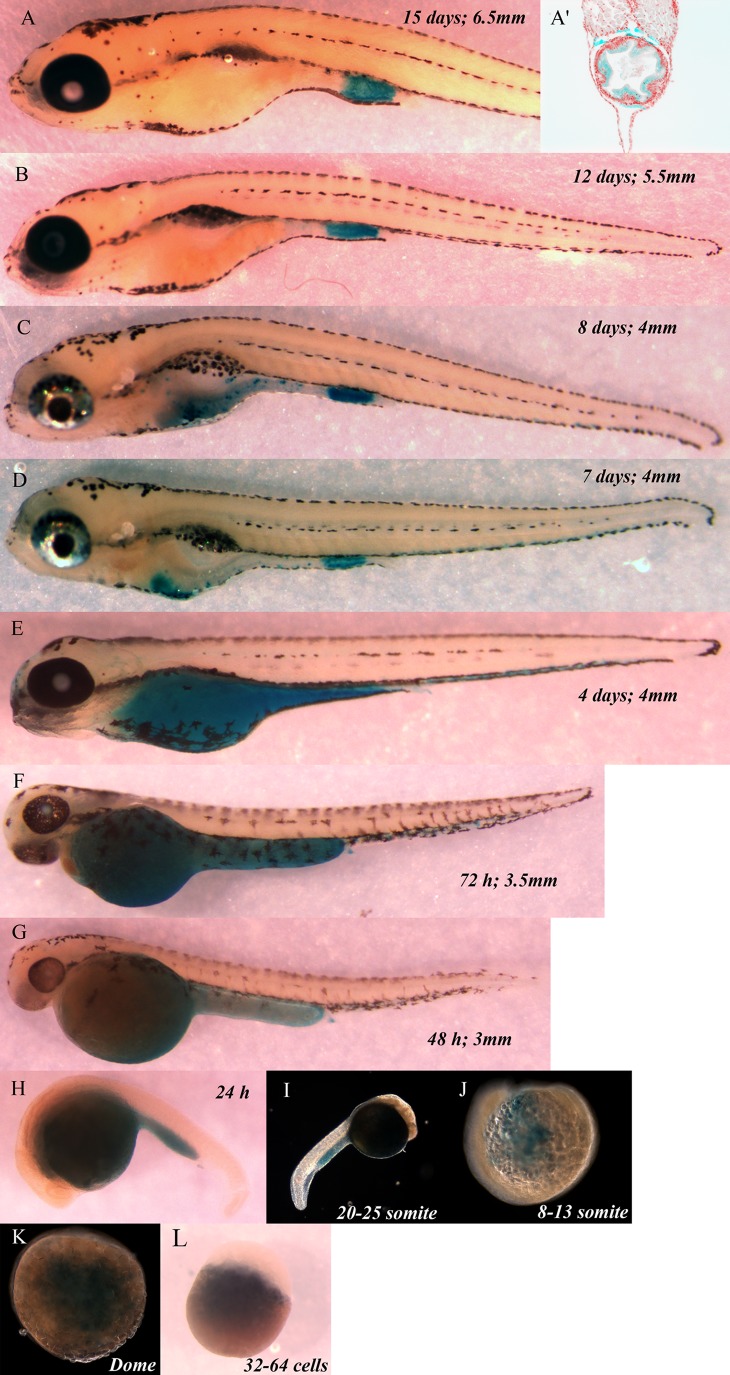
**Zebrafish stages, SaβG staining**. Presented from (A) 15 days post-fertilization animal; (A′) transverse section, cloaca level, DAPI staining of nuclei (red) and SaβG staining (blue); (B) 12 days; (C) 8 days; (D) 7 days; (E) 4 days; (F) 72 h; (G) 48 h; (H) 24 h; (I) 20-25 somite; (J) 8-13 somite; (K) Dome; and (L) 32-64 cells embryo. The length of the embryos is indicated in mm on each panel.

In order to further assess the evolutionary importance of senescence, developing zebrafish were stained for SaβG activity. SaβG staining is detected at very early stages including the yolk sac (Fig. 4), a temporary structure used as a source of energy for the developing embryo that is lost over time (Kimmel et al., 1995). The earliest detection of senescence in zebrafish is at the 32-64 cell stage (Fig. 4L). At the 8-13 somite stage senescence was detected in the yolk sac (Fig. 4J). From the 20-25 somite stage until 4 days post-fertilization senescence is detected in the yolk sac and in the tissues that will become the intestine (Fig. 4E-I). SaβG activity was previously reported in 3.5 day embryos (Kishi et al., 2008). From day 7 until day 15 senescence became restricted the cloacal end of the intestine (Fig. 4A-D). Our results thus suggest that senescence of the yolk sac is partly responsible for its elimination. At the moment we cannot fully rule out the possibility that some of the SaβG activity detected in developing zebrafish is related to autophagy. However, other studies which looked at senescence at specific time points in zebrafish observed identical SaβG activity to this study (Kishi et al., 2008), and this activity correlated with other markers associated with senescence such as p53 and p21 expressions (Donnini et al., 2010; Xia et al., 2014).

We did not detect any senescence signal in the zebrafish's pronephric area which may be due to the fact that bony fish have rudimentary kidneys all their life and therefore there is no need to eliminate them. Eliminating structures using cellular senescence provides opportunity for the surrounding tissues to replace or compensate the loss of those cells, which would benefit the morphogenetic process. In addition, the products secreted by senescent cells can stimulate cellular growth and migration of adjoining cells and thus have an impact on development. This indicates that signals associated with cellular senescence are highly conserved across a vast evolutionary distance among vertebrates from amphibians to mammals.

The discovery of senescence during embryonic development does not support the idea that it evolved for the sole purpose of protecting organisms from oncogenic transformation. It is more likely that it evolved primarily for embryonic development and is re-utilized in mature animals as a mechanism of tumor suppression. This report demonstrates that cellular senescence is present during the embryonic development of lower vertebrates (salamander and teleost fish). The paper by Yun's group (Davaapil et al., 2017) also shows that senescence is present in *Xenopus* as well as in axolotls. Our results, combined with the work by the groups of Yun (Davaapil et al., 2017), Serrano (Munoz-Espin et al., 2013) and Keyes (Storer et al., 2013), clearly demonstrate that senescence is a cellular process that evolved early in the vertebrate genealogy as it is present in organisms ranging from bony fish to amphibians all the way to mammals. Our work also suggests that developmental senescence is not associated with inflammation and displays a secretory pattern similar to mitochondrial dysfunction associated senescence (MiDAS) (Wiley et al., 2016). These novel observations open the door for new investigations on the evolutionary pressures shaping development and raises important questions about the evolution of molecular pathways for the selective induction of senescence during embryonic development.

## MATERIALS AND METHODS

### Senescence associated β-galactosidase

Embryos were fixed with 0.5% glutaraldehyde in PBS at 4°C 6 h to overnight and then rinsed two times for 15 min in PBS pH 5.5 containing 1 mM MgCl_2_ at 4°C. The embryos were stained for SaβG in X-gal staining solution (0.1% X-gal, 5 mM potassium ferrocyanide, 5 mM potassium ferricyanide, 150 mM Sodium chloride, and 2 mM magnesium chloride in PBS, pH 5.5) for 4-6 h at 37°C. Controls to confirm SaβG staining specificity were performed in identical conditions except the pH of the solutions were adjusted to 7.5 for which SaβG is inactive (none of the controls showed any sign of β-gal activity, *n*=10).

### Immunofluorescence enhanced with Tyramide

Sections were rehydrated as previously described then blocked using 2% BSA in TBS-T for 1 h at room temperature (Lévesque et al., 2010). Primary antibody was diluted in blocking solution (anti-p-Erk1/2, cat# 4370, Cell Signaling Technology, 1/400) and incubated on a slide overnight at 4°C. Secondary HRP coupled antibody was diluted (anti-rabbit HRP, cat# 170-6515, Bio-Rad, 1/800) in blocking solution for p-Erk1/2 and incubated at room temperature for 45 min. Tyramide (Biotium, San Francisco Bay, CA, cat# 92175) was diluted in 1× TBS with 0.0015% H_2_O_2_ to an active concentration of 11.6 µM then incubated at room temperature for 8 min. All slides were mounted with ProLong^®^ Gold antifade reagent containing DAPI (Invitrogen, cat# 36931). Slides were visualized with a Zeiss Axio Imager M2 Optical Microscope (Zeiss, Munich, Germany). The software used was the Zen 2 Pro Blue Edition (Zeiss, Munich, Germany, https://www.zeiss.com/microscopy/int/products/microscope-software/zen.html) with a Tile Module. All photos were verified using the range indicator of the software to make sure that they were not saturated. The photos were saved as TIF files and then imported into Photoshop CS4 (Adobe) to adjust the rotation and to crop the photos to be mounted into a multipanel figure using Illustrator CS4 (Adobe).

### Animal ethics conformity

Axolotl wild-type embryos were obtained from mating adult axolotls at the Université de Montréal. Zebrafish were generously provided by the laboratory of Dr Moldovan from the Sainte-Justine Hospital Research Institute affiliated with the Université de Montréal. All experiments and manipulation of animals were done in accordance with the requirement of the Université de Montréal animal ethics committee which is overseen by the Canadian Council of Animal Care.

### RNA isolation and qRT-PCR

Tissue samples were dissected from the pronephros region and tail region caudal to the pronephros (*n*=4). RNA was extracted with TRIzol reagent (Life Technologies, cat# 15596018) following the manufacturer's guidelines. RNA was quantified by diluting 1 µl of samples in 100 µl of 2.5× SYBR Green II (Life Technologies, cat# S7580), 5 mM Tris-HCl pH 8.0 and reading the fluorescence at excitation 485 nm and emission 520 nm. Average quantity of RNA obtained was 1 µg per sample. Reverse transcription was done using 200 ng of RNA. Reactions were done using SuperScript VILO™ (Life Technologies, cat# 11754-050) following the manufacturer's guidelines. 0.62 µM of oligo-dT17 was added to the mix. After completion of the reaction, the mix was diluted by adding 140 µl deionized water. qPCR reactions were done in 10 µl of 1× Sigma PCR Buffer (Sigma, cat# P2192-1VL), 2.5 mM MgCl_2_ (Sigma, cat# M1028-10×1 ml), 0.33X SYBR Green I (Life Technologies, cat# S7580), 20 µM dNTP, 250 nM each forward and reverse primers, 0.5 U JumpStart *taq* DNA polymerase (Sigma, cat# D9307-250Un) and 1 µl of sample cDNA. All primers were tested for their efficiency and are listed in Table S1. Cycling and measuring was performed using a Light Cycler 96 system (Roche, cat# 05815916001) with the following program: denaturation at 95°C 6 min, quantification with 50 cycles of 95°C 20 s, 58°C 20 s, 72°C 30 s, melting curve (95°C for 60 s, 40°C for 5 s, 65°C for 1 s up to 98°C by ramping at 0.07°C/s). Analysis was done using LightCycler 96 (software release 1.1.0.1320, Roche) and Cps were calculated using the ‘Abs Quant/2nd Derivative Max’ analysis. Expression is relative to GAPDH [normalization gene in salamanders limb regeneration (Vascotto et al., 2005)].
